# Does Neuroinflammation Underlie the Cognitive Changes Observed With Dietary Interventions?

**DOI:** 10.3389/fnins.2022.854050

**Published:** 2022-05-10

**Authors:** Jacqueline P. Robbins, Egle Solito

**Affiliations:** ^1^Barts and The London School of Medicine and Dentistry, Queen Mary University of London, London, United Kingdom; ^2^William Harvey Research Institute, Queen Mary University of London, London, United Kingdom

**Keywords:** ketogenic diet, calorie restriction, cognition, mood, neuroinflammation, neuroprotection, oxidative stress

## Abstract

Dietary interventions, such as calorie restriction and ketogenic diet, have been extensively studied in ageing research, including in cognitive decline. Epidemiological studies indicate beneficial effects of certain dietary regimes on mental health, including mood disorders and dementia. However, randomised-controlled trials (the gold-standard of evidence-based medicine) on calorie restriction diets and the ketogenic diet have yet to show clinically convincing effects in neuropsychiatric disorders. This review will examine the quality of studies and evidence base for the ketogenic and calorie restriction diets in common neuropsychiatric conditions, collating findings from preclinical experiments, case reports or small clinical studies, and randomised controlled clinical trials. The major cellular mechanisms that mediate the effects of these dietary interventions on brain health include neuroinflammation, neuroprotection, and neuromodulation. We will discuss the studies that have investigated the roles of these pathways and their interactions. Popularity of the ketogenic and calorie restriction diets has grown both in the public domain and in psychiatry research, allowing for informed review of the efficacy, the limitations, and the side effects of these diets in specific patient populations. In this review we will summarise the clinical evidence for these diets in neuropsychiatry and make suggestions to improve clinical translation of future research studies.

## Key Messages

- With increasing incidence of mental health problems and dementia worldwide, the potential of nutritional interventions including CR diets needs increased research efforts.
- Currently studies on CR and KD in neuropsychiatric disorders point to a modest beneficial effect on seizures, mood and cognitive symptoms. However, randomised controlled trials with large sample sizes are needed to determine the risk-benefit ratio and the clinical effect size with adequate certainty of the evidence.
- Research, monitoring and education for patients with co-morbidities, such as cardiovascular disease, IBD, or immunodeficiency are needed to prevent adverse events on physical or mental health when trialling these diets. Individuals with a medical condition should consult a dietician or doctor before trialling a CR or KD regime.

## Introduction

Diet is emerging as a key mediator of psychiatric disease, as it is to cardiology, endocrinology and other medical conditions (Sarris et al., 2015). Nonetheless, the vast majority of psychiatrists and other mental health professionals report no training in nutrition, despite all participants estimating the diets of their patients with mental health issues to be worse than the general population (Mörkl et al., 2021). All health professionals are pressured to advocate for healthy diets, but also compelled to practice evidence-based medicine. However, studies on nutrition in humans to date have been observational designs or small short-term interventions, rather than the randomised controlled trial (RCT) designs we demand in every other field of medicine (Schulze et al., 2018; Adan et al., 2019). The idea that healthy nutritious food is integral to our physical and mental wellbeing is so engrained that it complicates doing a placebo-controlled trial on mood. One may be biased to expecting a positive effect on a perceived healthy dietary arm and a negative effect on mood on an unhealthy arm. Therefore researching the impact of diet on mental health faces major challenges.

The association between diet and mental health is unequivocally accepted but investigating causality amongst the epidemiological data is problematic (Adan et al., 2019). There are multiple reasons for why mental health could affect diet. Firstly, long-term psychosocial factors could drive these results. People with a lower socioeconomic status are more likely to experience mental health problems, and people with mental health problems are more likely to be unemployed (Stansfeld et al., 2016), which can limit access to healthy and high-nutrient foods. Secondly, symptoms of severe mental illness such as low motivation, apathy and cognitive deficits could also affect dietary choices and access to healthcare. Thirdly, medications such as second-generation antipsychotics that affect appetite and cause metabolic complications are another contributing factor (Annamalai et al., 2017). Finally, in the short-term comfort eating may also play a role with psychological stress associated with higher consumption of unhealthy processed foods (Banta et al., 2019). These contributing variables mean it is now critical to have well-controlled intervention trials in-patient groups rather than solely observational studies to determine the effect of diet on mental health and cognition.

Obesity is associated with an increased risk for most of the major non-communicable diseases including diabetes, heart disease and Alzheimer’s disease; as well as a worse prognosis in COVID-19 (Nyberg et al., 2018; Paoli et al., 2020). The evidence linking chronic inflammation and obesity is increasing, and hence the interest in diet as a tool for improving health outcomes (Mattson, 2019). The Western diet, high in trans-fats and refined sugars, is associated with obesity, elevated oxidative stress and inflammation. The Western diet has been linked to cognitive impairment and mood disorders, with studies showing the hippocampus to be particularly vulnerable (Kanoski and Davidson, 2011). Dietary interventions of interest include the Mediterranean diet, anti-inflammatory diet, ketogenic diet and calorie restriction diets. The Mediterranean diet is rich in vegetables, grains, fish and unsaturated fats, and has strong evidence for its benefits in promoting metabolic, cardiovascular and mental health. These effects are thought to be mediated through its anti-inflammatory mechanisms and can be beneficial at any stage of illness (Mayr et al., 2018). There are multiple variations on the “anti-inflammatory” diet, but this is a permutation of the Mediterranean diet focusing on inclusion of polyunsaturated fatty acids and unprocessed carbohydrates with low glycaemic index. This has been studied in rheumatic disorders but has not been proven to reduce inflammatory markers (Zwickey et al., 2019). Due to individual variability and the multiple mechanisms through which diet affects our physiology it has been complex to determine the roles of different nutritional compounds on brain health.

In this review, we will restrict our focus to two popular diets the ketogenic diet (KD) and calorie restriction (CR), because they are thought primarily to act through the same mechanisms. Both diets result in an overall reduction in caloric intake and an increased level of circulating ketone bodies, beta-hydroxybutyrate (βHB) and acetoacetate. Intermittent fasting, also known as intermittent energy restriction, is the most widely used form of CR intervention as it is considered the most attainable for people compared to an overall daily reduction in calories. Both intermittent fasting and KDs result in higher ketone levels than a reduced daily food intake as in CR diets (Mattson et al., 2018). Table 1 summarises the major similarities and differences of CR and KD (Maalouf et al., 2009; Bok et al., 2019; de Cabo and Mattson, 2019).

**TABLE 1:** Comparison of the effects of the calorie restriction and ketogenic diets.

	Calorie restriction diet	Ketogenic diet
Physiological and metabolic changes	Reduction of glucose, elevation of ketone bodies	Elevation of ketone bodies, increase in fat oxidation, reduction of glucose
Physiological levels of ketones in humans (normal range < 0.6 mM)	βHB 0.2–0.8 mM in blood for overnight fasting (Pan et al., 2001; Burstal et al., 2018); βHB 2–3.5 mM in blood at 3 days of fasting (Hasselbalch et al., 1994; Pan et al., 2000)	βHB 0.4–5 mM in blood (Brehm et al., 2003; Anderson et al., 2021; Masino et al., 2021)
Key molecular mechanisms	Antioxidant effects, increased neurotrophic factors and neurogenesis, anti-inflammatory effects, inhibition of apoptosis	Antioxidant effects, anti-inflammatory effects, inhibition of apoptosis
Demonstrated beneficial effects in human neuropsychiatric disorders	Mild cognitive impairment, Alzheimer’s disease, depression	Schizophrenia, epilepsy, Alzheimer’s disease

The KD is a low carbohydrate/high lipid diet, where less than 10% of total daily calories should be obtained from carbohydrates, and over 70% from fats. It was originally proposed to treat medical conditions such as diabetes and epilepsy, but it has gained popularity for weight loss purposes. In this dietary programme the body is deprived of glucose for energy, necessitating a metabolic switch to utilising ketones as an energy source. In ketosis the ketone bodies are produced from lipids by the liver (Paoli et al., 2013). Supplementation of the diet with medium chain triglycerides (MCTs), ketone salts or ketone esters are other experimental method of establishing ketosis, without the need to strictly adhere to the KD (Cunnane et al., 2016).

**FIGURE 1:**
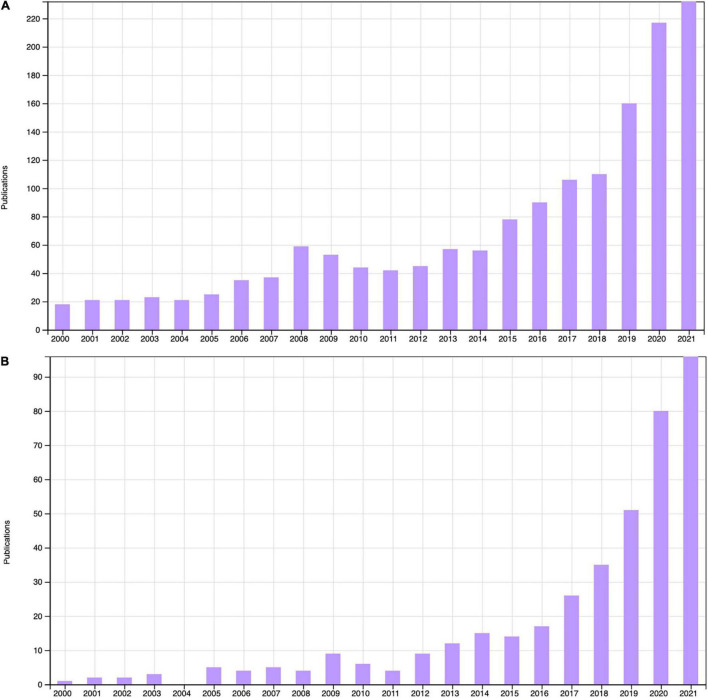
Peer-reviewed articles with “Ketogenic diet” in the title or abstract **(A)** and “Intermittent fasting” in the title or abstract **(B)** 2000–2021, Web of Science (accessed 16/12/2021).

CR and KD diets are rapidly growing in popularity. There are now over 1,500 publications with ketogenic diet in the title or abstract, and over 400 publications on intermittent fasting (Figure 1). Although the first publications on these diets were over 100 years ago intense investigation into their range of potential benefits and mechanisms has grown in the last twenty years. With growing public interest it is important to assess the evidence base for these diets in promoting cognitive and mood benefits, understand the mechanisms by which these act on the brain, and determine their relevance moving forward into large-scale clinical trials. This review will summarise key studies with relevance to clinical populations and discuss the limitations of these and considerations for study design going forward.

## Evidence for Intermittent Fasting and Ketogenic Diets in Neuropsychiatric Disorders

The brain is the most demanding organ for energy and the regulator of all energy intake and expenditure (Mattson, 2012). In a typical Western diet, the brain is dependent on glucose as the major energy source, despite the brain not having sufficient stores of this energy. In the absence of readily available glucose, such as during a strict CR or KD regime, the brain will switch to using ketones as its main energy source (Owen et al., 1967). βHB may provide a more efficient source of energy for brain per unit oxygen than glucose, although in the healthy brain it does not contribute meaningfully to energy production (Veech et al., 2001; Achanta and Rae, 2017). Nutritional ketosis, through a KD or supplemental MCTs has been demonstrated to be safe as it is usually self-limiting. A small increase in ketone plasma levels will stimulate insulin secretion, which in turn rapidly reduces plasma ketones (Owen et al., 1973); so nutritional ketosis does not induce ketoacidosis.

Intractable epilepsy in children led to the earliest use of fasting and KDs in neuropsychiatry, which has continued to be a mainstay of treatment for almost 100 years (Hartman et al., 2007). Numerous mechanisms have been proposed for how CR and ketosis may improve brain function, protecting against cognitive and mood decline. Animal studies have been critical for investigating whether these dietary interventions significantly affect behaviour and to identify the underlying mechanisms involved in these effects. The CR and KD have been reported to reduce systemic inflammation, increase the number and biogenesis of mitochondria, alter vasculature in the brain, regulate neurotransmitters and stimulate the expression of neurotrophic factors (Maalouf et al., 2007; Cunnane et al., 2016; Bok et al., 2019). Here we will look at the studies that have investigated the impact of CR or KD on neuropsychiatric disorders, from the preclinical animal models through to the clinical trials. The benefits of CR and KD for numerous disorders have come to be referred to as “facts” or “mainstays,” and it is now essential to critically analyse these studies on the quality of the evidence and the hope they offer for patients.

### Epilepsy

The KD has been a mainstay of treatment in intractable epilepsy in children for over 100 years, but the efficacy of the clinical studies needs to be evaluated based on today’s standards of evidence-based medicine. A systematic review found one-third of children with treatment-resistant epilepsy benefited from the KD with a reduction in seizure frequency of greater than 50% (Keene, 2006). The first RCT was performed for the KD in children with epilepsy in 2008, finding that 38% of children (out of 54 in the analysis) reported a greater than 50% reduction in seizures with KD compared to 6% in the standard diet arm (Neal et al., 2008). The Cochrane reviews of RCTs have found a promising antiepileptic efficacy for a ketogenic dietary plan with a high ratio of fat to carbohydrate, but that adverse side-effects and overall poor quality of the evidence needs to be considered (Martin K. et al., 2016; Martin-McGill et al., 2020). Evidence for an effect of KD in seizure control in adult epilepsy is extremely limited and there is need for a reliable RCT (Martin-McGill et al., 2020).

### Cognition

The high-energy availability of the Western style diet has been associated with cognitive impairments. Rodent studies have found that a high-fat diet with unrestricted food access (modelling the human Western diet) lead to poorer outcomes in learning and performance of cognitive tasks, with reduced brain-derived neurotrophic factor (BDNF) levels and lower protein density of the blood brain barrier (BBB) (Kanoski et al., 2007, 2010). The field of dietary restriction in ageing has numerous studies looking at how CR and KD during the rodent life span affects a range of behavioural functions; however, whether CR or KD robustly attenuates cognitive decline remains unclear (Ingram and de Cabo, 2017; Lilamand et al., 2020). Maze tasks testing effects of CR on cognition in ageing animals have produced a wide variety of results, suggesting that the genotype, age and sex of the animal all affect the outcome (Ingram et al., 1987; Means et al., 1993; Markowska and Savonenko, 2002; Ingram and de Cabo, 2017). Although the strength of the evidence for CR on improving cognition in ageing animals is variable, there are importantly no studies showing a detrimental effect of CR. Only one study showed worsening cognition when rats were maintained on severe CR at a weight of 280 g (approximately 35% below standard weight and inapplicable to human studies), and cognitive effects could be reversed by glucose administration (Yanai et al., 2004). The literature on the effect of the KD on cognition in preclinical studies shows a similar picture to CR, with eight studies to date of KD in animals testing models of cognition in ageing or Alzheimer’s disease. These studies showed improvement or no change of cognition and motor function in models of ageing, and importantly no adverse effects on lifespan or healthspan (Newman et al., 2017; Lilamand et al., 2020).

Clinical trials of effects of CR on cognition to date have not demonstrated clinically meaningful improvements. A 3-month regimen of 30% CR showed a positive effect on verbal memory in older adults (but not on working memory or attention tests), associated with reduced insulin and inflammatory marker CRP, but no change in serum IGF-1 or BDNF levels (Witte et al., 2009). CALERIE (Comprehensive Assessment of Long-term Effects of Reducing Intake of Energy) is a clinical trial investigating whether the positive findings of this diet in animal studies are replicated in humans. It started as a 6- and 12-month study with up to 30% CR in overweight individuals, extending to a 2-year phase 2 study to investigate 25% CR in 150 non-obese adults. No negative effects were found on mood, cognition, quality of life, sleep, and sexual function (Martin et al., 2007; Martin C. K. et al., 2016). This is an important result as previous studies on dieting and cognition in humans had shown an effect of diminished cognition, considered largely due to preoccupation with food (Redman and Ravussin, 2011). Cognitive testing found a small improvement on error rate during a spatial working memory test at 24 months only (Leclerc et al., 2020), but the small effect size in this small group has not been demonstrated to translate into any clinical significance and will need to be tested with clinically validated tools.

### Alzheimer’s Disease

The relationship between diet and neuropsychiatric symptoms can also be studied in terms of the effects of obesity on the brain. This is simpler in terms of studying real-world effects, although there is the risk of confounding diet and weight in this approach. Epidemiological studies find that obesity is associated with increased risk of dementia, with vascular risk factors further adding to the risk (Kivipelto et al., 2005). This was confirmed in a longitudinal study of ageing, showing the association between obesity and dementia risk to be independent of smoking, hypertension, diabetes, or major genetic risk factors (Ma et al., 2020). Hippocampal volume measured by MRI, which declines in old age and more rapidly in dementia, is also reduced in individuals with greater central obesity (Jagust et al., 2005).

Many mechanisms are proposed for how CR and KD may specifically benefit the brain in an Alzheimer’s disease state, including improving BBB integrity, increasing cerebral blood flow and increasing transport of β-amyloid (Aβ) (Paoli et al., 2019). Alzheimer’s disease is characterised by the abnormal aggregation of Aβ protein plaques and hyper-phosphorylated tau tangles; pathologies that have been widely reproduced in mouse models of the disease. Animal models of AD showed reduced Aβ plaque load in mice on KD (Van der Auwera et al., 2005) and squirrel monkeys on CR diets (Qin et al., 2006). Although middle age CR did not reduce Aβ plaques in macaques, it did reduce astrocyte activation (Sridharan et al., 2013; Bok et al., 2019). Only two studies to date (Aso et al., 2013; Kashiwaya et al., 2013) have looked at the cognitive benefits of KD in an animal model of Alzheimer’s disease, the latter demonstrating a beneficial effect on cognition and Aβ deposition in the brain.

Dysregulation of glucose metabolism and hyperinsulinemia have also been identified as early processes in Alzheimer’s disease prior to cognitive decline, causing a reduction in energy availability for neurons (Cunnane et al., 2016; Butterfield and Halliwell, 2019). Brain glucose uptake in older adults may be reduced by up to 8% in the frontal cortex (Nugent et al., 2014), an effect found to be more severe and widespread across the brain in Alzheimer’s disease (Castellano et al., 2015). Therefore, KD may have therapeutic potential in boosting available ATP in Alzheimer’s disease brain where oxidative phosphorylation and glucose metabolism are impaired. However, caution is needed here as induction of a calorie-restricted state in the brains of patients already deficient in available energy could have serious adverse outcomes. Transgenic APP mice were found to have an increased stress response and severe hypoglycaemia in response to CR, with aged mice especially sensitive to the dietary intervention and dying within days from hypoglycaemic shock (Pedersen et al., 1999). The length of dietary intervention and age of patient may be especially critical and will need to be determined in preclinical studies before large trials are conducted in this patient population.

The majority (eight out of nine) of the clinical studies on ketosis in MCI or AD have used a ketone supplementation approach with MCTs, rather than the typical KD. Six of these studies reported positive outcomes on cognitive assessments (Lilamand et al., 2020). The trial of MCT supplement AC-1202 (a compound containing caprylic acid found in coconut oil) was not found to improve cognition on the ADAS-Cog test in Alzheimer’s disease at 90 days. However, there were continued improvements at 90 days in a subgroup of patients who were not carriers of the Apolipoprotein-E ε4 gene allele, which could suggest that this approach may have benefits for a subgroup of patients (Henderson et al., 2009). A recent trial demonstrated feasibility of adherence to a KD in Alzheimer’s patients for a 12-week period with no adverse effects. Patients Addenbrookes Cognitive Examination (ACE) score and activities of daily living remained stable, compared to a decline in the control arm (Phillips et al., 2021).

### Mood Disorders

Although case reports have suggested a therapeutic benefit of the KD in individuals with major depressive disorder (MDD), bipolar disorder (BD) and schizophrenia, there are no RCTs of these disorders (Brietzke et al., 2018). Some animal studies support an antidepressant-like effect of the KD in models of depression. In a rodent study using “behavioural despair” induced immobility as the outcome, rats on a KD spent less time immobile than rats on a control diet, an effect that occurred without the need for rats to be in metabolic ketosis (Murphy et al., 2004). Key mechanisms of action proposed for KD overlap with hypotheses of contributory biological processes in mood disorders and schizophrenia: modulation of BDNF expression, mitochondrial dysfunction, and systemic inflammation (Brietzke et al., 2018; Sarnyai et al., 2019). Studies on the effects of these diets in humans with adequately powered sample sizes and validated mood and behavioural measures are needed in order to critically assess whether there is a therapeutic role for CR or KD in mood disorders.

Obesity and depression are highly comorbid disorders, both of which have been associated with chronic inflammation. Treatment resistance in mood disorders has also been associated with obesity and metabolic syndrome (Rizvi et al., 2014). Approximately 25% of patients with depression present with elevated peripheral inflammation (Osimo et al., 2019), but it is important to separate whether inflammation may be caused by the high rates of obesity in this group. McLaughlin et al. (2021) looked at overweight individuals with or without depression and matched them with healthy weight controls. They found that C-reactive protein (CRP) levels, a key plasma marker of inflammation, were higher in the overweight group with depression than in other groups (2.2 mg/L for overweight and depressed patients, 1.3 mg/L for overweight controls and 0.7 mg/L for normal weight and depressed). Weight loss interventions have been associated with reduced depressive symptoms and lower CRP levels in individuals with high BMI (Perez-Cornago et al., 2014), so there may be potential for CR diets to reduce mood symptoms and systemic inflammation.

#### Schizophrenia

The reduced life expectancy of people with schizophrenia has been established to be attributable to physical illness such as metabolic and cardiovascular disease, rather than a direct consequence of mental illness such as suicide. There are a number of factors contributing to this poor physical health, including side effects of antipsychotic medications, direct effect of the negative symptoms of schizophrenia (such as low motivation and neglect), and increased genetic risk of obesity and metabolic disease in this group (Annamalai et al., 2017). In 16 case-control studies, individuals with first-episode schizophrenia who were antipsychotic-naive were found already to have dysregulated glucose homeostasis (Pillinger et al., 2017). Evidence for brain glucose metabolism abnormalities in individuals with schizophrenia are demonstrated by magnetic resonance spectroscopy (Chouinard et al., 2017), proteomics and transcriptomics (Martins-de-Souza et al., 2011), and offer a sound rationale for exploring KD in this patient population. In the absence of novel antipsychotics without metabolic side effects, dietary and lifestyle interventions will be important in improving morbidity and mortality. Beyond this, it is also interesting to consider whether diet could play an additional role in improving symptoms of schizophrenia directly.

Human and rodent studies on the KD are too limited to make broad claims about its efficacy for this patient group (Bostock et al., 2017). In a NMDA receptor hypofunction mouse model of schizophrenia, 3 weeks of KD improved measures of psychomotor hyperactivity, stereotyped behaviour, social withdrawal and working memory deficits compared to a standard diet (Kraeuter et al., 2015). The KD protected prepulse inhibition of the startle reflex response, known to be deficient in schizophrenic patients and in the mouse model, as effectively as the antipsychotic olanzapine (Kraeuter et al., 2019). Especially relevant is that mice administered acute βHB showed improvement without KD adherence (Kraeuter et al., 2020).

Only one clinical study or case report of KD in schizophrenia to date had more than 2 patients: a study conducted in 1965 prior to the development of second-generation antipsychotics (reviewed by Bostock et al., 2017). Case studies of people diagnosed with schizophrenia or schizoaffective disorder trialling KD report reduction or resolution of positive symptoms, reversed when the diet is terminated or ketosis threshold is not maintained (Palmer, 2017). Gilbert-Jaramillo et al. (2018) trialled 6 weeks of the KD in a pair of twins with schizophrenia and found reductions in their Positive and Negative Symptom Scale (PANSS) scores and their BMI, despite neither patient adhering strictly to the diet due to its restrictiveness. KD could potentially be helpful in a wider population of people with schizophrenia through reducing the need or dosage of pharmacological treatments with adverse side effects. This is supported by case reports of patients on KD remaining free of psychotic symptoms after stopping medication (Palmer et al., 2019). The first RCT of KD in patients with bipolar disorder or schizophrenia will be completed later in 2022 (Sethi Dalai, ClinicalTrials.gov Identifier: NCT03935854).

## Mechanisms of Dietary Effect on the Brain

**FIGURE 2:**
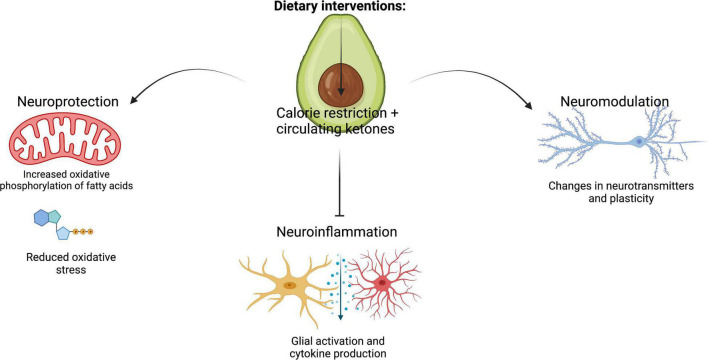
Summary of the key cellular mechanisms that mediate the effects of KD and CR on brain health: neuroinflammation, neuroprotection and neuromodulation. Figure created with BioRender.

KD and CR have been proposed to have multiple pathways through which they modulate neuronal cells, which can be broadly grouped into neuroprotection, neuromodulation and neuroinflammation (Figure 2).

### Neuroprotection

A popular theory is that CR induces an adaptive cellular stress response, activating specific transcription factors in this pathway (Calabrese et al., 2007). The regular activation of this pathway prepares the cells for future physiological stress, in a similar action to cardiovascular exercise training (de Cabo and Mattson, 2019). There are two main theories on how CR and KD may be neuroprotective: reduced production of reactive oxygen species (ROS) and increased production of neurotrophic factors (Maalouf et al., 2009).

Typically, mitochondria are the main source of ROS, causing oxidative damage. Mitochondria are critical for energy production in the brain, producing the majority of all ATP through oxidative phosphorylation. In Alzheimer’s disease the brain has a glucose deficit, shifting energy production away from glycolysis, in addition to a lowered mitochondrial bioenergetic capacity reducing ATP synthesis (Yao et al., 2009; Cunnane et al., 2016). These changes are associated with increased ROS production by the mitochondria causing oxidative damage. Ketones produce ATP via oxidative phosphorylation, and therefore this ATP source could be important for restoring the energy deficit in the Alzheimer’s disease brain. It is proposed KD and CR could reduce ROS in the brain, due to enhanced mitochondrial biogenesis and increased antioxidants (Maalouf et al., 2007, 2009). The KD upregulates metabolic and mitochondrial gene expression in the rodent hippocampus so could be especially beneficial in Alzheimer’s disease (Bough et al., 2006). Neurons, *in vitro*, that suffered from excitotoxic injury had significantly higher production of ROS by mitochondria, and treatment with ketones protected the neurons by increasing NADH oxidation in the mitochondria (Maalouf et al., 2007).

Increased levels of ketone bodies may be beneficial to neuroprotection, potentially without reducing energy intake. Acetoacetate has been shown to have anticonvulsant properties, and treatment with acetoacetate or acetone decreased seizure incidence in multiple mouse models of seizure (Rho et al., 2002; Likhodii et al., 2008). *In vitro* studies have offered evidence that this neuroprotection by exogenous ketone bodies could be through limiting glutamate excitotoxicity or oxidative stress. The reduced oxidative stress was found to be due to ketones increasing mitochondrial biogenesis whilst reducing ROS production, rather than due to increased antioxidant action (Maalouf et al., 2007, 2009).

Another mechanism of neuroprotection in CR may be through an increase in neurotrophic factor levels. Adult rats maintained on a restricted diet had increased expression of BDNF, which increased neurogenesis in the dentate gyrus (Lee et al., 2000). However, since the extent and relevance of neurogenesis is less clear in humans it is hard to extrapolate the importance of this action of neurotrophic factors. Another study found that BDNF levels were increased in the hippocampus, cerebral cortex and striatum in mice on an intermittent fasting regime, and that these mice were relatively protected from excitotoxicity, reversed when an antibody (Duan et al., 2001) blocked BDNF. Serum BDNF was found to increase in obese participants on a low calorie diet, suggesting dietary intake could be an important regulator of serum BDNF levels, and could be a relevant mechanism (Araya et al., 2008).

### Neuromodulation

Direct modulation of neuronal activity through changes in energy or neurotransmitter availability is another potential pathway for dietary effects on the CNS. The effect of ketosis in rodent models of epilepsy may act via a shift in the metabolism of glutamic acid leading to increased levels of inhibitory neurotransmitter gamma-aminobutryic acid (GABA) (Yudkoff et al., 2001). However, most studies of KD have not shown changes in GABA levels at a whole brain level, although regional changes could be a contributory mechanism (Hartman et al., 2007). Measuring neurotransmitter levels is challenging, but hippocampal transporters for glutamate and GABA were altered following KD in rats, and KD attenuated declining glutamate transporters in aged rats (Hernandez et al., 2018). Hori et al. (1992) investigated long-term potentiation, a cellular correlate of memory, in hippocampal slices of aged mice on a CR or *ad libitum* diet. They found aged CR mice had hippocampal neuronal profiles more similar to young mice of 2 months than to the age-matched mice fed *ad libitum*, suggesting another direct method for how diet could improve cognition. Conversely, rats on the KD showed reduced long-term potentiation, which may play a role in the increase in neuronal inhibition in children with epilepsy on the KD (Koranda et al., 2011). CR could also affect neurons of the hypothalamus through altered levels of appetite hormones. Leptin and insulin, anorexigenic hormones acting on the hypothalamus to control appetite, are found to be reduced following consumption of high fat diets. A high fat diet (unassociated with calorie load) caused a loss of neurons in the hypothalamus, demonstrating an interesting effect of diet and circulating hormones directly affecting neuronal cells within the CNS (Moraes et al., 2009).

### Neuroinflammation

Gut inflammation causing cytokine-mediated vagal activation, signalling to the hypothalamus and limbic system, is an established pathway in feeding regulation, sickness behaviours (such as low mood and social withdrawal) and pain perception. These signalling systems respond when there is an acute insult of gut homeostasis, such as exposure to a chemical or enterotoxigenic bacteria, triggering nausea, disgust, fatigue, and other brain-mediated responses. However, there are similar pathways affected during chronic perturbations to gut homeostasis, such as by different diets. These could utilise the vagal stimulation pathway, the stimulation of spinal afferent terminals or circumventricular organs lacking BBB protection, or the more widely explored system of cytokines crossing both the gastrointestinal blood barriers and blood brain barriers in systemic inflammation (Mayer, 2011).

All the pathways activated by CR and KD may directly or indirectly modulate neuroinflammatory processes. Direct effects could be through mitigating activation of glial cells, which can change from a resting to activated state in response to insult (such as by cytokines) and increase expression of inflammatory cytokines. Indirect regulation by CR could be through mediation of steroid hormones in HPA axis or through a reduction in circulating cytokine levels, which downstream reduce accumulation or aggregation of toxic proteins and ROS (Bok et al., 2019).

Support for the effect of inflammation in the gut on cognition and mood can be considered by looking at mental health in people with Inflammatory Bowel Disease (IBD). Psychiatric disorders such as depression and anxiety are present in almost one third of IBD patients, but the direction of causality for this co-morbidity is complex. However, behavioural disturbances are a risk factor before IBD diagnosis, and anti-inflammatory treatments of the gut are reported to improve mood and sleep (Collins, 2020). This suggests a need for more research to understand these mechanisms and patient groups whose mood may benefit from targeting systemic inflammation. Regarding dementia, a population-based cohort study following adults after their IBD diagnosis found that 5.5% of these patients developed dementia compared to 1.4% of controls matched for age, income and co-morbid conditions. Moreover, IBD patients developed dementia symptoms 7 years earlier on average (Zhang et al., 2021).

Animal studies have also looked at the effect of inflammation acting in the opposite direction, investigating a modulatory role of the brain on gut inflammation. The induction of depression caused a flare-up of colitis in a mouse model of IBD, with the stimulation of nicotinic acetylcholine receptors, disinhibiting vagal suppression of cytokines from the macrophages. Tricyclic antidepressants inhibited the reactivation of colitis, but only in the presence of depression in the mice (Ghia et al., 2009).

#### Blood-Brain-Barrier Permeability

The brain is considered an immune-privileged organ, with the BBB acting as a barrier to restrict access of immune cells and immune mediators to the CNS under normal conditions (Muldoon et al., 2013). The endothelial cells of the neurovasculature together with astrocytes and pericytes make up the major cell types of the BBB, which isolates the extracellular fluid of the CNS. Inflammation is known to affect the structural integrity of the BBB, leading to increased permeability, or a “leaky brain.” High fat diets modulate circulating inflammatory markers, disrupting the BBB structure by dysregulating production of tight junctions and basal lamina proteins, leading to neuroinflammation (Sheikh et al., 2022). Barrier breach can therefore result in both oxidative stress and cytokine stress within the CNS (Obrenovich, 2018). Long-term BBB dysfunction can consequently cause secondary activation of glial cells, neuronal dysfunction and degeneration (Wang et al., 2018; Sheikh et al., 2022). However, gliosis and cytokine production may also be causal to BBB damage via metabolic dysfunction, rather than a secondary event (Mauro et al., 2015).

A breakdown in the BBB has been linked with a range of neuropsychiatric disorders. Western-style high-energy diets affected cognition in a rat model, with reduced expression of tight junction proteins Claudin-5 and -12 in the choroid plexus and the BBB associated with impaired hippocampal-dependent learning (Kanoski et al., 2010). Microdissection of post-mortem tissue of schizophrenia patients showed evidence of a hypoinflammatory state in the BBB with molecular alterations of the cerebral microvasculature (Harris et al., 2008). However, clinical studies investigating the association between BBB permeability and Alzheimer’s pathology through the study of serum proteins in the CSF or brain have shown inconclusive results (Erickson and Banks, 2013).

#### Glial Cell Activation

When in a state of stress microglia switch into an activated state and are responsible for the phagocytic clearance of apoptotic neurons and other cells in the brain. This phagocytic process is important in maintaining brain homeostasis, but the pro-inflammatory state also causes microglia to produce cytokines, which are associated with neuronal damage (McArthur et al., 2010). Astrocytes also become activated in response to trauma and can act both by amplifying the inflammatory response of microglia and by secreting cytokines themselves (Bok et al., 2019). These are good defence mechanisms in acute stress, but under conditions of chronic stress to which the body is exposed in neurodegenerative disorders and other disease glial cell activation becomes damaging to neurons. Hormones critical to the regulation of appetite that act on the hypothalamus have been shown to exhibit inflammatory properties. Leptin (reduced in CR) has pro-inflammatory effects and promotes microglial activation, whereas corticosterone and ghrelin (increased in CR) have anti-inflammatory properties and suppress microglial activation (Radler et al., 2014).

Studies investigating the effects of CR on microglia report reduced expression of phagocytic markers and attenuated activation in the hypothalamus, but no change in microglia in other brain regions (Radler et al., 2014; Yin et al., 2018). Studies on astrocyte activation are limited, but middle-aged macaques given short-term CR showed a reduced rate of astrogliosis in the hippocampus (Sridharan et al., 2013).

#### Cytokine Levels

**FIGURE 3:**
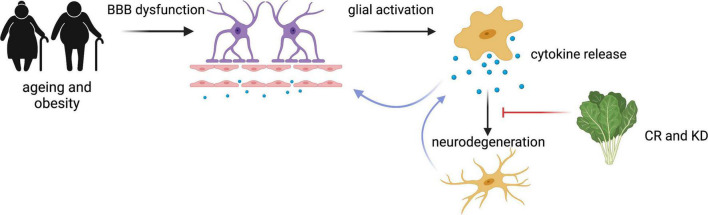
Summary of the neuroinflammatory processes mediated by CR and KD. Ageing and obesity states result in BBB dysfunction, glial activation and cytokine release, resulting in degeneration of neuronal cells and the further activation of glial cells (Wang et al., 2018; Sheikh et al., 2022). This process can be attenuated by CR (Hunt et al., 2006; Bok et al., 2019). Figure created with BioRender.

Neuroinflammatory pathways causing neuronal dysfunction described here are summarised in Figure 3. Although it is still under debate whether CR slows ageing and cognitive decline in mammals, it is widely accepted that it exerts anti-inflammatory effects (Balasubramanian et al., 2020; Dias et al., 2020). An inflammatory state, such as in Type 2 Diabetes, induces transcription of pro-inflammatory cytokines, including NF-κB, IL-1 beta, IL-6, TNFα (Sheikh et al., 2022). KD, CR and other low carbohydrate diets have been found to reduce circulating inflammatory markers (Chung et al., 2002; Forsythe et al., 2008; Dupuis et al., 2015), and suppress microglial activation in multiple brain regions (Horrillo et al., 2011). The pro-inflammatory cytokine NF-κB is expressed by all cells of the brain and is known to be involved in neuroinflammation through its regulation of glial cell activation (Mattson and Camandola, 2001). CR has been associated with reduced expression and phosphorylation activity of NF-κB in the brain (Mulrooney et al., 2011). It is reported that NF-κB could interact with ROS, and multiple studies have shown ROS activates NF-κB expression, an effect suppressed by a CR diet (Chung et al., 2002). βHB inhibits NF-κB-mediated inflammation, and ketone supplementation also suppresses cytokines IL-1β and IL-6 (Stubbs et al., 2020). The anti-inflammatory properties of the KD is now being investigated as a potential COVID-19 therapy to determine if the KD attenuates the cytokine storm in severe COVID-19 and reduce ICU admissions (Samir, ClinicalTrials.gov Identifier: NCT04492228).

#### The Microbiome-Gut-Brain Axis

The microbiome may also be a central mechanism regulating the potential pathways through which diet affects brain function, including via immunomodulation, vagus nerve signalling and tryptophan metabolism (Cryan and Dinan, 2012). The human gut microbiota describes the trillions of microbial and bacterial species that colonise the gastrointestinal tract (Gill et al., 2006). Dysregulation of the microbiome may be a cause or consequence of inflammation in the gut and is suggested to affect the BBB (Obrenovich et al., 2017). High-fibre diets have been found to encourage microbiomes that generate short chain fatty acids (SCFAs) to thrive, with both a direct contribution to barrier function and a positive effect on colonocytes of the gut (Braniste et al., 2014). Conversely the Western diet does not favour these microbiota species, but supports bacteria species associated with chronic inflammation (Paoli et al., 2019). Microbial metabolites, such as the methylamine trimethylamine-N-oxide, have been shown to promote BBB function and integrity, and improve working memory test performance (Hoyles et al., 2021).

As early as 1910, George Porter Phillips published his study on lactic acid bacteria and their effect on symptoms of depression on his patients in the Bethlem Royal Hospital (London-United Kingdom). For his study he switched his patients to a meat-free diet supplemented with a kefir drink and reported a significant improvement of mood symptoms in 13/18 patients (Phillips, 1910). Possible benefits of fermented foods have since been shown in depression and anxiety in a small proportion of patients, and may depend on inflammatory status (Aslam et al., 2020). Germ-free mice that lack a gut microbiome are a good model system for studying the effects of the microbiome in a controlled environment, independent of effects on the brain due to dietary nutrients. Microglia numbers in the brain have been reported to be lower in germ-free mice, although overall neuron and astrocyte numbers are unchanged, and these microglia had a reduced range in their innate immune response. The altered microglial phenotype is also found in antibiotic-treated mice and restored by adding SCFAs to the diet (Erny et al., 2015). A meta-analysis of 68 studies investigating prebiotics and synbiotics, which are fermented by the microbiota to produce SCFAs, found promising evidence supporting anti-inflammatory benefits, but there was vast heterogeneity of findings between these human studies (McLoughlin et al., 2017).

Other proposed methods of action for the microbiome are through the direct production of neurotransmitters, indirect neuromodulation, and mediation of the secretory activity of the HPA axis. Chronic administration with a *Lactobacillus* species in mice modulated GABA expression, reduced stress-induced corticosterone levels, and dampened anxiety- and depression-related behaviours (Bravo et al., 2011). Germ-free mice also had raised plasma corticosterone in response to restraint stress, with this HPA axis stress response reversible with introduction of *Bifidobacterium infantis* bacteria. c-Fos activation in the hypothalamus preceded changes in plasma cytokine levels, suggesting both neuromodulatory and neuroinflammatory pathways at play (Sudo et al., 2004). Lower levels of glutamate and higher levels of GABA were found in the hippocampi of mice receiving faecal transplants from schizophrenia patients compared to microbiome transplants from healthy controls (Zheng et al., 2019).

Only a handful of studies have investigated the link between the KD and gut microbiome to date, but studies in both human and mice showed an overall decrease in the diversity of microbiota species (Paoli et al., 2019). A study on seizure activity in mice found an overall decrease in microbiota species, but a specific increase in two bacteria that mediated an anti-seizure effect. This study proposes that the overall reduction in microbiota species and colonisation of 2 KD-associated bacteria may provide the mechanism for the therapeutic effect of the KD in epilepsy (Olson et al., 2018). Supplementation of a high fat diet with a ketone ester also reduced microbiota diversity, demonstrating that this effect is due to the presence of βHB, rather than fat (Ang et al., 2020).

## Barriers to Implementation of Dietary Interventions

### Limitations of Clinical Studies

Preclinical studies have been hampered by inconsistencies between species, genetic strains, sex, age of animals and the dietary intervention protocol. Ensuring impact of future clinical trials will require rigorous planning of study designs. The first major consideration is participant recruitment. Larger trials are needed to draw conclusions that can be translated to mainstream clinical practice. This requires both larger numbers of participants and larger numbers of trial centres due to issues of researcher bias between centres in existing studies (Bostock et al., 2017; Martin-McGill et al., 2020). Stratifying participants by gene alleles or co-morbidities may also be important to understand which groups these interventions are most effective for. The AC-1202 trial in Alzheimer’s disease only showed benefit in APOE-ε3 carriers (Henderson et al., 2009), and CALERIE reported reduced CRP with CR, which may be useful for specific population subgroups with raised inflammatory markers (Redman and Ravussin, 2011). Moreover, some co-morbidities may interfere with mechanisms of CR, and some may be criteria for exclusion. For example, detrimental effects of CR have been reported in preclinical studies such as immune response to infection, wound healing and decline in bone health, which may make some patients high-risk (Ingram and de Cabo, 2017). Recruiting patients with similar diets prior to the trial is another consideration that may reduce confounding factors (as proved by Li et al., 2019).

Planning of study design and analysis is also critical. Ideally RCTs would be double-blinded, although blinding participants or carers is problematic with diets, and this is an advantage of MCT supplements (Keene, 2006; Henderson et al., 2009). Remarkably few studies measured blood ketone levels or transparently reported these (see Table 1), which will be essential for future meta-analyses and for individuals with at-home monitoring. The length of dietary intervention is another variable. Previous studies in children with epilepsy have reported that KD efficacy can be determined after just 3 months, with no further increase by 6 months, and children who discontinued the KD in the first year maintained a statistically significant long-term improvement, offering reassurance for both researchers and parents in future trials (Marsh et al., 2006; Wu et al., 2016). David et al. (2014) reported rapid changes in microbiome composition of healthy volunteers after just 5 days of switching to a plant-based or animal-based diet, and it would be important to determine whether clinically relevant effects on mood and cognition can be achieved in a matter of weeks rather than years. Standardisation across dietary protocols coordinated by dieticians would ensure safe nutrient balances and allow efficacy to be determined across studies (Wahl et al., 2017). Final analysis considerations include the importance of controlling for weight loss to investigate the effect of metabolic switching alone, and whether efficacy varies between participants of different BMIs.

### Limitations of Calorie Restriction and Ketogenic Diet

The concept of eating three meals a day is engrained into our society, and many people are unwilling to fast or to eat precise, calorie-measured portions long-term (Adan et al., 2019). The abundance of food, and the central role of sharing meals in our society and in cultural celebrations also make it difficult to strictly adhere to these restrictive diets. Early studies on starvation by Johns Hopkins University in the 1960s did not realise the effect that small levels of carbohydrate intake by participants (such as from cola drinks) had on their study, until analysing their urinary nitrogen excretion results (Owen, 2005). Analysis of trial results largely relies on participants accurately self-reporting what they ate over the trial, which is complex and error-prone. The CALERIE study on 25% CR found it only achieved 12% reduction in calories at 24 months by participants, demonstrating the difficulty most people find with CR adherence. The KD was hard to adhere to for longer than 2 weeks due to severe cravings for high sugar foods and the difficulty of partaking in social events (Gilbert-Jaramillo et al., 2018). This study was conducted over the Christmas festive period, and so timing of future trials must account for cultural and dietary customs. Studies during religious periods of fasting could be considered although changes to medication timings must also be factored in. However, the positive news from these studies is the potential for efficacy even without strict adherence, making this intervention more applicable to a wider population.

Poor tolerability remains the major challenge for both the study and the roll out of these diets in patient groups. Initial side effects in these trials are commonly a lack of concentration due to preoccupation with food. However, patients can be counselled that these typically disappear within 1 month (Redman and Ravussin, 2011; de Cabo and Mattson, 2019). CALERIE phase 2 had no serious adverse events reported, but more than 20% of participants reported dysmenorrhoea in the CR arm, which may require women to be followed up in clinic more carefully. The KD has been especially hampered by reports of adverse events, and children with epilepsy typically stopped the intervention due to frequent vomiting, constipation and diarrhoea (Martin-McGill et al., 2020). Hyperlipidaemia and slowed growth are also long-term concerns in this young patient group and it is important to monitor children on this diet closely (Maalouf et al., 2009). It has been proposed future trials use the Modified Atkins Diet, which is more palatable with fewer side effects (Martin-McGill et al., 2020). In adults on KD strict adherence can cause halitosis, gastrointestinal complaints, muscle cramps, headaches and nephrolithiasis (O’Neill and Raggi, 2020). Ketone supplements, such as MCTs, may be beneficial in reducing the serious side-effects associated with the KD, but it is worth noting that in the trial of AC-1202 in Alzheimer’s disease patients there was a discontinuation rate of 23% due to adverse events, mostly diarrhoea, flatulence, and dyspepsia (Henderson et al., 2009). It will be important to fully understand the potential harms before advising clinicians to recommend these diets to patients. Qualitative data is also essential to understand what aspects of these dietary interventions patients and carers struggle with if we are to promote adherence beyond the clinical trial setting.

## Discussion

Reviewing the cellular mechanisms underlying the effects of these diets on cognition and mental health demonstrates how closely intertwined these processes of inflammation, oxidative stress, mitochondrial function and neuronal function are, and how typically a perturbation in one pathway will affect the other pathways. This is supported by clinical trial data, with reduced markers for energy expenditure, tissue oxidative damage, pro-inflammatory cytokine TNFα and inflammatory marker CRP in the CR group of the CALERIE trial after 24 months (Dorling et al., 2021). Since these diets affect multiple interlinked pathways it is important to understand the risk-benefit ratio of committing to them long-term before recommending them in a clinical setting. It is also important to understand these pathways in different disorders, to ensure interventional studies do not have adverse consequences on brain function.

At present it is difficult to assess whether modulating inflammation in the patient populations discussed would help in replicating or enhancing the therapeutic effects of CR, and additional research into the inflammatory status of these patients is warranted. CR and KD may help in subgroups of patients where systemic inflammation is detected, but may be less effective in patients with low baseline inflammation. Further research focused on understanding the mechanisms may also help elucidate therapeutic interventions that initiate the same benefits as CR and KD without the difficulties associated with maintaining a strict diet (Gasior et al., 2006).

More rigorous RCTs are needed to understand the benefits of these diets, ideally with study variables being standardised, especially a standardised diet programme. The age at which CR or KD need to be commenced, and the length and extent of adherence that is required for a clinically meaningful effect is essential to determine. It is exciting that previous studies have shown positive outcomes in participants that have only adhered to the dietary interventions for varying time periods or discontinued early, such as in trials for epilepsy and cognition. This information could be very important in achieving a minimum level of concordance in trial participants and encouraging patients to attempt a new therapeutic strategy even if they are unsure of their ability to fully commit. It has been proposed that time-restricted feeding may offer benefits independently of any overall CR, and so may be preferable in populations wanting to avoid weight loss or other potential adverse impacts (Ingram and de Cabo, 2017; Balasubramanian et al., 2020). More research into the safety and tolerability of these diets in patient groups is essential due to the common frequency of side effects discussed earlier. With the chronic inflammation and increased risk for disease clinicians should advocate for transitioning away from the Western diet, however until larger clinical studies are published it is not possible to advocate for either the intermittent fasting or KD over other healthy eating diets such as the Mediterranean diet.

### Future Directions

Exogenous ketone supplements, such as MCTs, that induce nutritional ketosis pathways are an attractive avenue to offer benefits of KD without the strict dietary adherence. Studies on these so far have shown similar outcomes to dietary manipulation in models of epilepsy and Alzheimer’s disease, and may be able to offer the same neuroprotective effects without any calorie restriction. Henderson et al. (2009) show that blood ketone levels of 0.5 mM resulted in beneficial effects, a similar level as in KD (see Table 1). Rodent models offer a method to study the threshold for ketosis with ketone supplementation in developmental conditions such as epilepsy and autism where restrictive diets will be problematic to implement. These studies show sustained ketosis with ketone supplementation added to a normal diet, reduced seizure activity (Ciarlone et al., 2016; Kovács et al., 2019), reduced anxiety-like behaviours (Ari et al., 2017; Hollis et al., 2018), improved performance on cognitive tasks, and enhanced hippocampal synaptic plasticity (Ciarlone et al., 2016). Dogs with idiopathic epilepsy on anti-seizure medications showed an overall minor reduction of seizures with MCT supplementation into a standard diet, but demonstrated safety alongside medications and no adverse effects (Berk et al., 2020). Although pre-clinical studies suggest supplements had fewer side effects than the diets, one clinical trial that reported on adverse events noted a similar rate of dropouts due to side effects with MCT supplementation as found in KD trials (Henderson et al., 2009). A comprehensive understanding of the mechanisms that CR and KD act through will be critical to identify and test compounds and supplements that can induce the positive outcomes of the diets without the difficulties of adhering to them over a long time. With ketone supplements and home monitoring devices entering the consumer market this will be an interesting branch of lifestyle medicine to follow. Improving palatability of these supplements will be another important challenge for this field.

Another promising intervention could be the manipulation of microbiomes by prebiotic supplements, which are metabolised by probiotic bacteria in the gut. Although more research is needed on how the microbiome affects the brain, prebiotics could be directly beneficial as a monotherapy or could be a useful supplement alongside the KD, which has been found to reduce microbiota diversity (Paoli et al., 2019). Research into the extent of the role of the gut-brain-axis could also be essential for improving the impacts of chronic inflammation on mood in people living with IBD (Mayer, 2011).

## Conclusion

The preclinical *in vivo*, small clinical study and larger clinical trial data for CR and KD have been reviewed here and all support a potential benefit for CR and KD diets in several psychiatric disorders, but the quality of evidence is still low. For the contemporary field of nutritional psychiatry to establish a robust evidence-base, a shift from observational and epidemiological studies to interventional and reproducible RCTs is now essential (Adan et al., 2019). Clinicians have little training in diet and nutrition, despite this being a great public health challenge and a key driver of disease. The Mörkl et al. (2021) study suggests that mental health professionals would like more training in nutrition, however, the evidence-based interventions for this teaching are still lacking.

Finally, interventional and epidemiological studies on diet and nutrition demonstrate that healthy eating patterns are associated with good mental health (Firth et al., 2020). As clinicians we need to act on this knowledge and offer better food to our patients in inpatient units, and by extension in any healthcare setting. There is now enough evidence for us to know this could have positive outcomes for some patient groups, and there is no evidence to suggest any undesirable effects. Wider policy implementation, guided by specific studies on mental health and dementia prevention, are needed to promote healthy eating and lifestyle at a national level.

## Author Contributions

JR and ES planned manuscript scope. JR drafted the manuscript. ES revised drafts and final proofs. Both authors contributed to the article and approved the submitted version.

## Conflict of Interest

The authors declare that the research was conducted in the absence of any commercial or financial relationships that could be construed as a potential conflict of interest.

## Publisher’s Note

All claims expressed in this article are solely those of the authors and do not necessarily represent those of their affiliated organizations, or those of the publisher, the editors and the reviewers. Any product that may be evaluated in this article, or claim that may be made by its manufacturer, is not guaranteed or endorsed by the publisher.

## References

[B1] Achanta L. B. Rae C. D. (2017). β-Hydroxybutyrate in the brain: one molecule, multiple mechanisms. *Neurochem. Res.* 42 35–49. 10.1007/s11064-016-2099-2 27826689

[B2] Adan R. A. van der Beek E. M. Buitelaar J. K. Cryan J. F. Hebebrand J. Higgs S. (2019). Nutritional psychiatry: towards improving mental health by what you eat. *Eur. Neuropsychopharmacol.* 29 1321–1332. 10.1016/j.euroneuro.2019.10.011 31735529

[B3] Anderson J. C. Mattar S. G. Greenway F. L. Lindquist R. J. (2021). Measuring ketone bodies for the monitoring of pathologic and therapeutic ketosis. *Obes. Sci. Pract.* 7 646–656. 10.1002/osp4.516 34631141 PMC8488448

[B4] Ang Q. Y. Alexander M. Newman J. C. Tian Y. Cai J. Upadhyay V. (2020). Ketogenic diets alter the gut microbiome resulting in decreased intestinal Th17 cells. *Cell* 181 1263–1275. 10.1016/j.cell.2020.04.027 32437658 PMC7293577

[B5] Annamalai A. Kosir U. Tek C. (2017). Prevalence of obesity and diabetes in patients with schizophrenia. *World J. Diabetes* 8:390. 10.4239/wjd.v8.i8.390 28861176 PMC5561038

[B6] Araya A. V. Orellana X. Espinoza J. (2008). Evaluation of the effect of caloric restriction on serum BDNF in overweight and obese subjects: preliminary evidences. *Endocrine* 33 300–304. 10.1007/s12020-008-9090-x 19012000

[B7] Ari C. Kovács Z. Juhasz G. Murdun C. Goldhagen C. R. Koutnik A. P. (2017). Exogenous ketone supplements reduce anxiety-related behavior in Sprague-Dawley and Wistar Albino Glaxo/Rijswijk rats. *Front. Mol. Neurosci.* 9:137. 10.3389/fnmol.2016.00137 PMC5138218 27999529

[B8] Aslam H. Green J. Jacka F. N. Collier F. Berk M. Pasco J. (2020). Fermented foods, the gut and mental health: a mechanistic overview with implications for depression and anxiety. *Nutr. Sci.* 23 659–671. 10.1080/1028415x.2018.1544332 30415609

[B9] Aso E. Semakova J. Joda L. Semak V. Halbaut L. Calpena A. (2013). Triheptanoin supplementation to ketogenic diet curbs cognitive impairment in APP/PS1 mice used as a model of familial Alzheimer’s disease. *Curr. Alzheimer Res.* 10 290–297. 10.2174/15672050112099990128 23131121

[B10] Balasubramanian P. DelFavero J. Ungvari A. Papp M. Tarantini A. Price N. (2020). Time-restricted feeding (TRF) for prevention of age-related vascular cognitive impairment and dementia. *Ageing Res. Rev.* 64:101189. 10.1016/j.arr.2020.101189 32998063 PMC7710623

[B11] Banta J. E. Segovia-Siapco G. Crocker C. B. Montoya D. Alhusseini N. (2019). Mental health status and dietary intake among California adults: a population-based survey. *Int. J. Food sci. Nutr.* 70 759–770. 10.1080/09637486.2019.1570085 30773065

[B12] Berk B. A. Law T. H. Packer R. M. Wessmann A. Bathen-Nöthen A. Jokinen T. S. (2020). A multicenter randomized controlled trial of medium-chain triglyceride dietary supplementation on epilepsy in dogs. *J. Vet. Int. Med.* 34 1248–1259. 10.1111/jvim.15756 32293065 PMC7255680

[B13] Bok E. Jo M. Lee S. Lee B. R. Kim J. Kim H. J. (2019). Dietary restriction and neuroinflammation: a potential mechanistic link. *Int. J. Mol. Sci.* 20:464. 10.3390/ijms20030464 30678217 PMC6386998

[B14] Bostock E. Kirkby K. C. Taylor B. V. (2017). The current status of the ketogenic diet in psychiatry. *Front. Psychiatry* 8:43. 10.3389/fpsyt.2017.00043 28373848 PMC5357645

[B15] Bough K. J. Wetherington J. Hassel B. Pare J. F. Gawryluk J. W. Greene J. G. (2006). Mitochondrial biogenesis in the anticonvulsant mechanism of the ketogenic diet. *Ann. Neurol.* 60 223–235. 10.1002/ana.20899 16807920

[B16] Braniste V. Al-Asmakh M. Kowal C. Anuar F. Abbaspour A. Tóth M. (2014). The gut microbiota influences blood-brain barrier permeability in mice. *Sci. Transl. Med.* 6 ra158–ra263. 10.1126/scitranslmed.3009759 25411471 PMC4396848

[B17] Bravo J. A. Forsythe P. Chew M. V. Escaravage E. Savignac H. M. Dinan T. G. (2011). Ingestion of Lactobacillus strain regulates emotional behavior and central GABA receptor expression in a mouse via the vagus nerve. *Proc. Natl. Acad. Sci. U.S.A.* 108 16050–16055. 10.1073/pnas.1102999108 21876150 PMC3179073

[B18] Brehm B. J. Seeley R. J. Daniels S. R. D’Alessio D. A. (2003). A randomized trial comparing a very low carbohydrate diet and a calorie-restricted low fat diet on body weight and cardiovascular risk factors in healthy women. *J. Clin. Endocrinol. Metab.* 88 1617–1623. 10.1210/jc.2002-021480 12679447

[B19] Brietzke E. Mansur R. B. Subramaniapillai M. Balanzá-Martínez V. Vinberg M. González-Pinto A. (2018). Ketogenic diet as a metabolic therapy for mood disorders: evidence and developments. *Neurosci. Biobehav. Rev.* 94 11–16. 10.1016/j.neubiorev.2018.07.020 30075165

[B20] Burstal R. J. Reilly J. R. Burstal B. (2018). Fasting or starving? Measurement of blood ketone levels in 100 fasted elective and emergency adult surgical patients at an Australian tertiary hospital. *Anaesthes. Intens. Care* 46 463–467. 10.1177/0310057X1804600506 30189819

[B21] Butterfield D. A. Halliwell B. (2019). Oxidative stress, dysfunctional glucose metabolism and Alzheimer disease. *Nat. Rev. Neurosci.* 20 148–160. 10.1038/s41583-019-0132-6 30737462 PMC9382875

[B22] Calabrese E. J. Bachmann K. A. Bailer A. J. Bolger P. M. Borak J. Cai L. (2007). Biological stress response terminology: integrating the concepts of adaptive response and preconditioning stress within a hormetic dose–response framework. *Toxicol. Appl. Pharmacol.* 222 122–128. 10.1016/j.taap.2007.02.015 17459441

[B23] Castellano C. A. Nugent S. Paquet N. Tremblay S. Bocti C. Lacombe G. (2015). Lower brain 18F-Fluorodeoxyglucose uptake but normal 11C-Acetoacetate metabolism in mild Alzheimer’s Disease dementia. *J. Alzheimers Dis.* 43 1343–1353. 10.3233/JAD-141074 25147107

[B24] Chouinard V. A. Kim S. Y. Valeri L. Yuksel C. Ryan K. P. Chouinard G. (2017). Brain bioenergetics and redox state measured by 31P magnetic resonance spectroscopy in unaffected siblings of patients with psychotic disorders. *Schizophr. Res.* 187 11–16. 10.1016/j.schres.2017.02.024 28258794 PMC5581291

[B25] Chung H. Y. Kim H. J. Kim K. W. Choi J. S. Yu B. P. (2002). Molecular inflammation hypothesis of aging based on the anti-aging mechanism of calorie restriction. *Microsc. Res. Tech.* 59 264–272. 10.1002/jemt.10203 12424787

[B26] Ciarlone S. L. Grieco J. C. D’Agostino D. P. Weeber E. J. (2016). Ketone ester supplementation attenuates seizure activity, and improves behavior and hippocampal synaptic plasticity in an Angelman syndrome mouse model. *Neurobiol. Dis.* 96 38–46. 10.1016/j.nbd.2016.08.002 27546058

[B27] Collins S. M. (2020). Interrogating the gut-brain axis in the context of inflammatory bowel disease: a translational approach. *Inflamm. Bowel Dis.* 26 493–501. 10.1093/ibd/izaa004 31970390 PMC7054772

[B28] Cryan J. F. Dinan T. G. (2012). Mind-altering microorganisms: the impact of the gut microbiota on brain and behaviour. *Nat. Rev. Neurosci.* 13 701–712. 10.1038/nrn3346 22968153

[B29] Cunnane S. C. Courchesne-Loyer A. Vandenberghe C. St-Pierre V. Fortier M. Hennebelle M. (2016). Can ketones help rescue brain fuel supply in later life? Implications for cognitive health during aging and the treatment of Alzheimer’s disease. *Front. Mol. Neurosci.* 9:53. 10.3389/fnmol.2016.00053 27458340 PMC4937039

[B30] David L. A. Maurice C. F. Carmody R. N. Gootenberg D. B. Button J. E. Wolfe B. E. (2014). Diet rapidly and reproducibly alters the human gut microbiome. *Nature* 505 559–563. 10.1038/nature12820 24336217 PMC3957428

[B31] de Cabo R. Mattson M. P. (2019). Effects of intermittent fasting on health, aging, and disease. *N. Engl. J. Med.* 381 2541–2551. 10.1056/nejmra1905136 31881139

[B32] Dias I. R. de Sousa Santos C. de Oliveira L. R. S. Peixoto M. F. D. De Sousa R. A. L. Cassilhas R. C. (2020). Does calorie restriction improve cognition? *IBRO Rep.* 9 37–45. 10.1016/j.ibror.2020.05.001 33336102 PMC7733132

[B33] Dorling J. L. van Vliet S. Huffman K. M. Kraus W. E. Bhapkar M. Pieper C. F. (2021). Effects of caloric restriction on human physiological, psychological, and behavioral outcomes: highlights from CALERIE phase 2. *Nutr. Rev.* 79 98–113. 10.1093/nutrit/nuaa085 32940695 PMC7727025

[B34] Duan W. Guo Z. Mattson M. P. (2001). Brain-derived neurotrophic factor mediates an excitoprotective effect of dietary restriction in mice. *J. Neurochem.* 76 619–626. 10.1046/j.1471-4159.2001.00071.x 11208925

[B35] Dupuis N. Curatolo N. Benoist J. F. Auvin S. (2015). Ketogenic diet exhibits anti-inflammatory properties. *Epilepsia* 56 e95–e98. 10.1111/epi.13038 26011473

[B36] Erickson M. A. Banks W. A. (2013). Blood–brain barrier dysfunction as a cause and consequence of Alzheimer’s disease. *J. Cereb. Blood Flow Metab.* 33 1500–1513. 10.1038/jcbfm.2013.135 23921899 PMC3790938

[B37] Erny D. de Angelis A. L. H. Jaitin D. Wieghofer P. Staszewski O. David E. (2015). Host microbiota constantly control maturation and function of microglia in the CNS. *Nat. Neurosci.* 18 965–977. 10.1038/nn.4030 26030851 PMC5528863

[B38] Firth J. Gangwisch J. E. Borisini A. Wootton R. E. Mayer E. A. (2020). Food and mood: how do diet and nutrition affect mental wellbeing? *BMJ* 2020:369. 10.1136/bmj.m2382 PMC7322666 32601102

[B39] Forsythe C. E. Phinney S. D. Fernandez M. L. Quann E. E. Wood R. J. Bibus D. M. (2008). Comparison of low fat and low carbohydrate diets on circulating fatty acid composition and markers of inflammation. *Lipids* 43 65–77. 10.1007/s11745-007-3132-7 18046594

[B40] Gasior M. Rogawski M. A. Hartman A. L. (2006). Neuroprotective and disease-modifying effects of the ketogenic diet. *Behav. Pharmacol.* 17:431. 10.1097/00008877-200609000-00009 16940764 PMC2367001

[B41] Ghia J. E. Blennerhassett P. Deng Y. Verdu E. F. Khan W. I. Collins S. M. (2009). Reactivation of inflammatory bowel disease in a mouse model of depression. *Gastroenterology* 136 2280–2288. 10.1053/j.gastro.2009.02.069 19272381

[B42] Gilbert-Jaramillo J. Vargas-Pico D. Espinosa-Mendoza T. Falk S. Llanos-Fernández K. Guerrero-Haro J. (2018). The effects of the ketogenic diet on psychiatric symptomatology, weight and metabolic dysfunction in schizophrenia patients. *Clin. Nutr. Metab.* 1 1–5. 10.1093/med/9780197501207.003.0001

[B43] Gill S. R. Pop M. DeBoy R. T. Eckburg P. B. Turnbaugh P. J. Samuel B. S. (2006). Metagenomic analysis of the human distal gut microbiome. *Science* 312 1355–1359. 10.1126/science.1124234 16741115 PMC3027896

[B44] Harris L. W. Wayland M. Lan M. Ryan M. Giger T. Lockstone H. (2008). The cerebral microvasculature in schizophrenia: a laser capture microdissection study. *PLoS One* 3:e3964. 10.1371/journal.pone.0003964 19088852 PMC2597747

[B45] Hartman A. L. Gasior M. Vining E. P. Rogawski M. A. (2007). The neuropharmacology of the ketogenic diet. *Pediatr. Neurol.* 36 281–292. 10.1016/j.pediatrneurol.2007.02.008 17509459 PMC1940242

[B46] Hasselbalch S. G. Knudsen G. M. Jakobsen J. Hageman L. P. Holm S. Paulson O. B. (1994). Brain metabolism during short-term starvation in humans. *J. Cereb. Blood Flow Metab.* 14 125–131. 10.1038/jcbfm.1994.17 8263048

[B47] Henderson S. T. Vogel J. L. Barr L. J. Garvin F. Jones J. J. Costantini L. C. (2009). Study of the ketogenic agent AC-1202 in mild to moderate Alzheimer’s disease: a randomized, double blind, placebo-controlled, multicenter trial. *Nutr. Metab.* 6 1–25. 10.1186/1743-7075-6-31 19664276 PMC2731764

[B48] Hernandez A. R. Hernandez C. M. Campos K. T. Truckenbrod L. M. Sakarya Y. McQuail J. A. (2018). The antiepileptic ketogenic diet alters hippocampal transporter levels and reduces adiposity in aged rats. *J. Gerontol. A* 73 450–458. 10.1093/gerona/glx193 29040389 PMC5861916

[B49] Hollis F. Mitchell E. S. Canto C. Wang D. Sandi C. (2018). Medium chain triglyceride diet reduces anxiety-like behaviors and enhances social competitiveness in rats. *Neuropharmacology* 138 245–256. 10.1016/j.neuropharm.2018.06.017 29908242

[B50] Hori N. Hirotsu I. Davis P. J. Carpenter D. O. (1992). Long-term potentiation is lost in aged rats but preserved by calorie restriction. *Neuroreport* 3 1085–1088. 10.1097/00001756-199212000-00013 1337284

[B51] Horrillo D. Sierra J. Arribas C. García-San Frutos M. Carrascosa J. M. Lauzurica N. (2011). Age-associated development of inflammation in Wistar rats: effects of caloric restriction. *Arch. Physiol. Biochem.* 117 140–150. 10.3109/13813455.2011.577435 21635187

[B52] Hoyles L. Pontifex M. G. Rodriguez-Ramiro I. Anis-Alavi M. A. Jelane K. S. Snelling T. (2021). Regulation of blood–brain barrier integrity by microbiome-associated methylamines and cognition by trimethylamine N-oxide. *Microbiome* 9 1–21. 10.1186/s40168-021-01181-z 34836554 PMC8626999

[B53] Hunt N. D. Hyun D. H. Allard J. S. Minor R. K. Mattson M. P. Ingram D. K. (2006). Bioenergetics of aging and calorie restriction. *Ageing Res. Rev.* 5 125–143. 10.1007/978-90-481-8556-6_8 16644290

[B54] Ingram D. K. de Cabo R. (2017). Calorie restriction in rodents: caveats to consider. *Ageing Res. Rev.* 39 15–28. 10.1016/j.arr.2017.05.008 28610949 PMC5565679

[B55] Ingram D. K. Weindruch R. Spangler E. L. Freeman J. R. Walford R. L. (1987). Dietary restriction benefits learning and motor performance of aged mice. *J. Gerontol.* 42 78–81. 10.1093/geronj/42.1.78 3794202

[B56] Jagust W. Harvey D. Mungas D. Haan M. (2005). Central obesity and the aging brain. *Arch. Neurol.* 62 1545–1548. 10.1001/archneur.62.10.1545 16216937

[B57] Kanoski S. E. Davidson T. L. (2011). Western diet consumption and cognitive impairment: links to hippocampal dysfunction and obesity. *Physiol. Behav.* 103 59–68. 10.1016/j.physbeh.2010.12.003 21167850 PMC3056912

[B58] Kanoski S. E. Meisel R. L. Mullins A. J. Davidson T. L. (2007). The effects of energy-rich diets on discrimination reversal learning and on BDNF in the hippocampus and prefrontal cortex of the rat. *Behav. Brain Res.* 182 57–66. 10.1016/j.bbr.2007.05.004 17590450 PMC2042136

[B59] Kanoski S. E. Zhang Y. Zheng W. Davidson T. L. (2010). The effects of a high-energy diet on hippocampal function and blood-brain barrier integrity in the rat. *J. Alzheimer Dis.* 21 207–219. 10.3233/JAD-2010-091414 20413889 PMC4975946

[B60] Kashiwaya Y. Bergman C. Lee J. H. Wan R. King M. T. Mughal M. R. (2013). A ketone ester diet exhibits anxiolytic and cognition-sparing properties, and lessens amyloid and tau pathologies in a mouse model of Alzheimer’s disease. *Neurobiol. Aging* 34 1530–1539. 10.1016/j.neurobiolaging.2012.11.023 23276384 PMC3619192

[B61] Keene D. L. (2006). A systematic review of the use of the ketogenic diet in childhood epilepsy. *Pediatr. Neurol.* 35 1–5. 10.1016/j.pediatrneurol.2006.01.005 16814077

[B62] Kivipelto M. Ngandu T. Fratiglioni L. Viitanen M. Kåreholt I. Winblad B. (2005). Obesity and vascular risk factors at midlife and the risk of dementia and Alzheimer disease. *Arch. Neurol.* 62 1556–1560. 10.1001/archneur.62.10.1556 16216938

[B63] Koranda J. L. Ruskin D. N. Masino S. A. Blaise J. H. (2011). A ketogenic diet reduces long-term potentiation in the dentate gyrus of freely behaving rats. *J. Neurophysiol.* 106 662–666. 10.1152/jn.00001.2011 21613596 PMC3154820

[B64] Kovács Z. D’Agostino D. P. Diamond D. M. Ari C. (2019). Exogenous ketone supplementation decreased the lipopolysaccharide-induced increase in absence epileptic activity in Wistar Albino Glaxo Rijswijk rats. *Front. Mol. Neurosci.* 12:45. 10.3389/fnmol.2019.00045 30930744 PMC6427924

[B65] Kraeuter A. K. Archambault N. van den Buuse M. Sarnyai Z. (2019). Ketogenic diet and olanzapine treatment alone and in combination reduce a pharmacologically-induced prepulse inhibition deficit in female mice. *Schizophr. Res.* 212 221–224. 10.1016/j.schres.2019.08.002 31405622

[B66] Kraeuter A. K. Loxton H. Lima B. C. Rudd D. Sarnyai Z. (2015). Ketogenic diet reverses behavioral abnormalities in an acute nmda receptor hypofunction model of schizophrenia. *Schizophr. Res.* 169 491–493. 10.1016/j.schres.2015.10.041 26547882

[B67] Kraeuter A. K. Mashavave T. Suvarna A. van den Buuse M. Sarnyai Z. (2020). Effects of beta-hydroxybutyrate administration on MK-801-induced schizophrenia-like behaviour in mice. *Psychopharmacology* 237 1397–1405. 10.1007/s00213-020-05467-2 31993694

[B68] Leclerc E. Trevizol A. P. Grigolon R. B. Subramaniapillai M. McIntyre R. S. Brietzke E. (2020). The effect of caloric restriction on working memory in healthy non-obese adults. *CNS Spectr.* 25 2–8. 10.1017/S1092852918001566 30968820

[B69] Lee J. Duan W. Long J. M. Ingram D. K. Mattson M. P. (2000). Dietary restriction increases the number of newly generated neural cells, and induces BDNF expression, in the dentate gyrus of rats. *J. Mol. Neurosci.* 15 99–108. 10.1385/JMN:15:2:99 11220789

[B70] Li B. He Y. Ma J. Huang P. Du J. Cao L. (2019). Mild cognitive impairment has similar alterations as Alzheimer’s disease in gut microbiota. *Alzheimers Dement.* 15 1357–1366. 10.1016/j.jalz.2019.07.002 31434623

[B71] Likhodii S. Nylen K. Burnham W. M. (2008). Acetone as an anticonvulsant. *Epilepsia* 49 83–86. 10.1111/j.1528-1167.2008.01844.x 19049597

[B72] Lilamand M. Porte B. Cognat E. Hugon J. Mouton-Liger F. Paquet C. (2020). Are ketogenic diets promising for Alzheimer’s disease? A translational review. *Alzheimers Res. Ther.* 12 1–10. 10.1186/s13195-020-00615-4 32290868 PMC7158135

[B73] Ma Y. Ajnakina O. Steptoe A. Cadar D. (2020). Higher risk of dementia in English older individuals who are overweight or obese. *Int. J. Epidemiol.* 49 1353–1365. 10.1093/ije/dyaa099 32575116 PMC7660153

[B74] Maalouf M. Rho J. M. Mattson M. P. (2009). The neuroprotective properties of calorie restriction, the ketogenic diet, and ketone bodies. *Brain Res. Rev.* 59 293–315. 10.1016/j.brainresrev.2008.09.002 18845187 PMC2649682

[B75] Maalouf M. Sullivan P. G. Davis L. Kim D. Y. Rho J. M. (2007). Ketones inhibit mitochondrial production of reactive oxygen species production following glutamate excitotoxicity by increasing NADH oxidation. *Neuroscience* 145 256–264. 10.1016/j.neuroscience.2006.11.065 17240074 PMC1865572

[B76] Markowska A. L. Savonenko A. (2002). Retardation of cognitive aging by life-long diet restriction: implications for genetic variance. *Neurobiol. Aging* 23 75–86. 10.1016/s0197-4580(01)00249-4 11755022

[B77] Marsh E. B. Freeman J. M. Kossoff E. H. Vining E. P. Rubenstein J. E. Pyzik P. L. (2006). The outcome of children with intractable seizures: a 3-to 6-year follow-up of 67 children who remained on the ketogenic diet less than one year. *Epilepsia* 47 425–430. 10.1111/j.1528-1167.2006.00439.x 16499771

[B78] Martin C. K. Anton S. D. Han H. York-Crowe E. Redman L. M. Ravussin E. (2007). Examination of cognitive function during six months of calorie restriction: results of a randomized controlled trial. *Rejuvenation Res.* 10 179–190. 10.1089/rej.2006.0502 17518698 PMC2664681

[B79] Martin C. K. Bhapkar M. Pittas A. G. Pieper C. F. Das S. K. Williamson D. A. (2016). Effect of calorie restriction on mood, quality of life, sleep, and sexual function in healthy nonobese adults: the CALERIE 2 randomized clinical trial. *JAMA Int.Med.* 176 743–752. 10.1001/jamainternmed.2016.1189 PMC4905696 27136347

[B80] Martin K. Jackson C. F. Levy R. G. Cooper P. N. (2016). Ketogenic diet and other dietary treatments for epilepsy. *Cochrane Database Syst. Rev.* 2:CD001903. 26859528 10.1002/14651858.CD001903.pub3

[B81] Martin-McGill K. J. Bresnahan R. Levy R. G. Cooper P. N. (2020). Ketogenic diets for drug-resistant epilepsy. *Cochrane Database Syst. Rev.* 2020:6. 10.1002/14651858.CD001903.pub5 PMC7387249 32588435

[B82] Martins-de-Souza D. Harris L. W. Guest P. C. Bahn S. (2011). The role of energy metabolism dysfunction and oxidative stress in schizophrenia revealed by proteomics. *Antioxid. Redox Signal.* 15 2067–2079. 10.1089/ars.2010.3459 20673161

[B83] Masino S. A. Ruskin D. N. Freedgood N. R. Lindefeldt M. Dahlin M. (2021). Differential ketogenic diet-induced shift in CSF lipid/carbohydrate metabolome of pediatric epilepsy patients with optimal vs. no anticonvulsant response: a pilot study. *Nutr. Metab.* 18 1–11. 10.1186/s12986-020-00524-1 33648550 PMC7923458

[B84] Mattson M. P. (2012). Energy intake and exercise as determinants of brain health and vulnerability to injury and disease. *Cell Metab.* 16 706–722. 10.1016/j.cmet.2012.08.012 23168220 PMC3518570

[B85] Mattson M. P. (2019). An evolutionary perspective on why food overconsumption impairs cognition. *Trends Cogn. Sci.* 23 200–212. 10.1016/j.tics.2019.01.003 30670325 PMC6412136

[B86] Mattson M. P. Camandola S. (2001). NF-κB in neuronal plasticity and neurodegenerative disorders. *J. Clin. Invest.* 107 247–254. 10.1172/jci11916 11160145 PMC199201

[B87] Mattson M. P. Moehl K. Ghena N. Schmaedick M. Cheng A. (2018). Intermittent metabolic switching, neuroplasticity and brain health. *Nat. Rev. Neurosci.* 19:63. 10.1038/nrn.2017.156 PMC5913738 29321682

[B88] Mauro C. De Rosa V. Marelli-Berg F. Solito E. (2015). Metabolic syndrome and the immunological affair with the blood–brain barrier. *Front. Immunol.* 5:677. 10.3389/fimmu.2014.00677 25601869 PMC4283608

[B89] Mayer E. A. (2011). Gut feelings: the emerging biology of gut–brain communication. *Nat. Rev. Neurosci.* 12 453–466. 10.1038/nrn3071 21750565 PMC3845678

[B90] Mayr H. L. Tierney A. C. Thomas C. J. Ruiz-Canela M. Radcliffe J. Itsiopoulos C. (2018). Mediterranean-type diets and inflammatory markers in patients with coronary heart disease: a systematic review and meta-analysis. *Nutr. Res.* 50 10–24. 10.1016/j.nutres.2017.10.014 29540268

[B91] McArthur S. Cristante E. Paterno M. Christian H. Roncaroli F. Gillies G. E. (2010). Annexin A1: a central player in the anti-inflammatory and neuroprotective role of microglia. *J. Immunol.* 185 6317–6328. 10.4049/jimmunol.1001095 20962261 PMC3145124

[B92] McLaughlin A. P. Nikkheslat N. Hastings C. Nettis M. A. Kose M. Worrell C. (2021). The influence of comorbid depression and overweight status on peripheral inflammation and cortisol levels. *Psychol. Med.* 18 1–8. 10.1017/S0033291721000088 33731235 PMC9693673

[B93] McLoughlin R. F. Berthon B. S. Jensen M. E. Baines K. J. Wood L. G. (2017). Short-chain fatty acids, prebiotics, synbiotics, and systemic inflammation: a systematic review and meta-analysis. *Am. J. Clin. Nutr.* 106 930–945. 10.3945/ajcn.117.156265 28793992

[B94] Means L. W. Higgins J. L. Fernandez T. J. (1993). Mid-life onset of dietary restriction extends life and prolongs cognitive functioning. *Physiol. Behav.* 54 503–508. 10.1016/0031-9384(93)90243-9 8415944

[B95] Moraes J. C. Coope A. Morari J. Cintra D. E. Roman E. A. Pauli J. R. (2009). High-fat diet induces apoptosis of hypothalamic neurons. *PLoS One* 4:e5045. 10.1371/journal.pone.0005045 19340313 PMC2661137

[B96] Mörkl S. Stell L. Buhai D. V. Schweinzer M. Wagner-Skacel J. Vajda C. (2021). ‘An Apple a Day’?: psychiatrists. psychologists and psychotherapists report poor literacy for nutritional medicine: international survey spanning 52 countries. *Nutrients* 13:822. 10.3390/nu13030822 33801454 PMC8000813

[B97] Muldoon L. L. Alvarez J. I. Begley D. J. Boado R. J. Del Zoppo G. J. Doolittle N. D. (2013). Immunologic privilege in the central nervous system and the blood–brain barrier. *J. Cereb. Blood Flow Metab.* 33 13–21. 10.1038/jcbfm.2012.153 23072749 PMC3597357

[B98] Mulrooney T. J. Marsh J. Urits I. Seyfried T. N. Mukherjee P. (2011). Influence of caloric restriction on constitutive expression of NF-κB in an experimental mouse astrocytoma. *PLoS One* 6:e18085. 10.1371/journal.pone.0018085 21479220 PMC3068150

[B99] Murphy P. Likhodii S. Nylen K. Burnham W. M. (2004). The antidepressant properties of the ketogenic diet. *Biol. Psychiatry* 56 981–983. 10.1016/j.biopsych.2004.09.019 15601609

[B100] Neal E. G. Chaffe H. Schwartz R. H. Lawson M. S. Edwards N. Fitzsimmons G. (2008). The ketogenic diet for the treatment of childhood epilepsy: a randomised controlled trial. *Lancet Neurol.* 7 500–506. 10.1016/S1474-4422(08)70092-9 18456557

[B101] Newman J. C. Covarrubias A. J. Zhao M. Yu X. Gut P. Ng C. P. (2017). Ketogenic diet reduces midlife mortality and improves memory in aging mice. *Cell Metab.* 26 547–557. 10.1016/j.cmet.2017.08.004 28877458 PMC5605815

[B102] Nugent S. Castellano C. A. Goffaux P. Whittingstall K. Lepage M. Paquet N. (2014). Glucose hypometabolism is highly localized but lower cortical thickness and brain atrophy are widespread in cognitively normal older adults. *Am. J. Physiol. Endocrinol. Metab.* 306 E1315–E1321. 10.1152/ajpendo.00067.2014 24735889

[B103] Nyberg S. T. Batty G. D. Pentti J. Virtanen M. Alfredsson L. Fransson E. I. (2018). Obesity and loss of disease-free years owing to major non-communicable diseases: a multicohort study. *Lancet Public Health* 3 e490–e497. 10.1016/S2468-2667(18)30139-7 30177479 PMC6178874

[B104] Obrenovich M. E. (2018). Leaky gut, leaky brain? *Microorganisms* 6:107. 10.3390/microorganisms6040107 PMC6313445 30340384

[B105] Obrenovich M. Rai H. Mana T. S. Shola D. McCloskey B. Sass C. (2017). Dietary co-metabolism within the microbiota-gut-brain-endocrine metabolic interactome. *BAO Microbiol* 2:22. 10.2174/1871527315666160202123107 26831263

[B106] Olson C. A. Vuong H. E. Yano J. M. Liang Q. Y. Nusbaum D. J. Hsiao E. Y. (2018). The gut microbiota mediates the anti-seizure effects of the ketogenic diet. *Cell* 173 1728–1741. 10.1016/j.cell.2018.04.027 29804833 PMC6003870

[B107] O’Neill B. Raggi P. (2020). The ketogenic diet: pros and cons. *Atherosclerosis* 292 119–126. 10.1016/j.atherosclerosis.2019.11.021 31805451

[B108] Osimo E. F. Baxter L. J. Lewis G. Jones P. B. Khandaker G. M. (2019). Prevalence of low-grade inflammation in depression: a systematic review and meta-analysis of CRP levels. *Psychol. Med.* 49 1958–1970. 10.1017/S0033291719001454 31258105 PMC6712955

[B109] Owen O. E. (2005). Ketone bodies as a fuel for the brain during starvation. *Biochem. Mol. Biol. Educ.* 33 246–251. 10.1002/bmb.2005.49403304246

[B110] Owen O. E. Morgan A. P. Kemp H. G. Sullivan J. M. Herrera M. G. Cahill G. J. (1967). Brain metabolism during fasting. *J. Clin. Invest.* 46 1589–1595. 10.1172/JCI105650 6061736 PMC292907

[B111] Owen O. E. Reichard G. A. Jr. Markus H. Boden G. Mozzoli M. A. Shuman C. R. (1973). Rapid intravenous sodium acetoacetate infusion in man. Metabolic and kinetic responses. *J. Clin. Invest.* 52 2606–2616. 10.1172/JCI107453 4729054 PMC302521

[B112] Palmer C. M. (2017). Ketogenic diet in the treatment of schizoaffective disorder: two case studies. *Schizophr. Res.* 189 208–209. 10.1016/j.schres.2017.01.053 28162810

[B113] Palmer C. M. Gilbert-Jaramillo J. Westman E. C. (2019). The ketogenic diet and remission of psychotic symptoms in schizophrenia: two case studies. *Schizophr. Res.* 208 439–440. 10.1016/j.schres.2019.03.019 30962118

[B114] Pan J. W. Rothman D. L. Behar K. L. Stein D. T. Hetherington H. P. (2000). Human brain β-hydroxybutyrate and lactate increase in fasting-induced ketosis. *J. Cereb. Blood Flow Metab.* 20 1502–1507. 10.1097/00004647-200010000-00012 11043913

[B115] Pan J. W. Telang F. W. Lee J. H. De Graaf R. A. Rothman D. L. Stein D. T. (2001). Measurement of β−hydroxybutyrate in acute hyperketonemia in human brain. *J. Neurochem.* 79 539–544. 10.1046/j.1471-4159.2001.00575.x 11701757

[B116] Paoli A. Gorini S. Caprio M. (2020). The dark side of the spoon-glucose, ketones and COVID-19: a possible role for ketogenic diet? *J. Transl. Med.* 18 1–9. 10.1186/s12967-020-02600-9 33218357 PMC7677746

[B117] Paoli A. Mancin L. Bianco A. Thomas E. Mota J. F. Piccini F. (2019). Ketogenic diet and microbiota: friends or enemies? *Genes* 10:534. 10.3390/genes10070534 31311141 PMC6678592

[B118] Paoli A. Rubini A. Volek J. S. Grimaldi K. A. (2013). Beyond weight loss: a review of the therapeutic uses of very-low-carbohydrate (ketogenic) diets. *Eur. J. Clin. Nutr.* 67:789. 10.1038/ejcn.2013.116 23801097 PMC3826507

[B119] Pedersen W. A. Culmsee C. Ziegler D. Herman J. P. Mattson M. P. (1999). Aberrant stress response associated with severe hypoglycemia in a transgenic mouse model of Alzheimer’s disease. *J. Mol. Neurosci.* 13 159–165. 10.1385/JMN:13:1-2:159 10691302

[B120] Perez-Cornago A. de la Iglesia R. Lopez-Legarrea P. Abete I. Navas-Carretero S. Lacunza C. I (2014). A decline in inflammation is associated with less depressive symptoms after a dietary intervention in metabolic syndrome patients: a longitudinal study. *Nutr. J.* 13 1–9. 10.1186/1475-2891-13-36 24762259 PMC4013804

[B121] Phillips J. G. P. (1910). The treatment of melancholia by the lactic acid Bacillus. *Br. J. Psychiatry* 56 422–431. 10.1192/bjp.56.234.422

[B122] Phillips M. C. Deprez L. M. Mortimer G. M. Murtagh D. K. McCoy S. Mylchreest R. (2021). Randomized crossover trial of a modified ketogenic diet in Alzheimer’s disease. *Alzheimers Res. Ther.* 13 1–12. 10.1186/s13195-021-00783-x 33622392 PMC7901512

[B123] Pillinger T. Beck K. Gobjila C. Donocik J. G. Jauhar S. Howes O. D. (2017). Impaired glucose homeostasis in first-episode schizophrenia: a systematic review and meta-analysis. *JAMA Psychiatry* 74 261–269. 10.1001/jamapsychiatry.2016.3803 28097367 PMC6352957

[B124] Qin W. Chachich M. Lane M. Roth G. Bryant M. de Cabo R. (2006). Calorie restriction attenuates Alzheimer’s disease type brain amyloidosis in Squirrel monkeys (*Saimiri sciureus*). *J. Alzheimers Dis.* 10 417–422. 10.3233/jad-2006-10411 17183154

[B125] Radler M. E. Hale M. W. Kent S. (2014). Calorie restriction attenuates lipopolysaccharide (LPS)-induced microglial activation in discrete regions of the hypothalamus and the subfornical organ. *Brain Behav. Immunity* 38 13–24. 10.1016/j.bbi.2013.11.014 24291211

[B126] Redman L. M. Ravussin E. (2011). Caloric restriction in humans: impact on physiological, psychological, and behavioral outcomes. *Antioxid. Redox Signal.* 14 275–287. 10.1089/ars.2010.3253 20518700 PMC3014770

[B127] Rho J. M. Anderson G. D. Donevan S. D. White H. S. (2002). Acetoacetate, acetone, and dibenzylamine (a contaminant in L-(+)−β−hydroxybutyrate) exhibit direct anticonvulsant actions in vivo. *Epilepsia* 43 358–361. 10.1046/j.1528-1157.2002.47901.x 11952765

[B128] Rizvi S. J. Grima E. Tan M. Rotzinger S. Lin P. McIntyre R. S. (2014). Treatment-resistant depression in primary care across Canada. *Can. J. Psychiatry* 59 349–357. 10.1177/070674371405900702 25007419 PMC4086317

[B129] Sarnyai Z. Kraeuter A. K. Palmer C. M. (2019). Ketogenic diet for schizophrenia: clinical implication. *Curr. Opin. Psychiatry* 32 394–401. 10.1097/YCO.0000000000000535 31192814

[B130] Sarris J. Logan A. C. Akbaraly T. N. Amminger G. P. Balanzá-Martínez V. Freeman M. P. (2015). Nutritional medicine as mainstream in psychiatry. *Lancet Psychiatry* 2 271–274. 10.1016/s2215-0366(14)00051-0 26359904

[B131] Schulze M. B. Martínez-González M. A. Fung T. T. Lichtenstein A. H. Forouhi N. G. (2018). Food based dietary patterns and chronic disease prevention. *Br. J. Med.* 361:k2396. 10.1136/bmj.k2396 29898951 PMC5996879

[B132] Sheikh M. H. Errede M. d’Amati A. Khan N. Q. Fanti S. Loiola R. A. (2022). Impact of metabolic disorders on the structural, functional, and immunological integrity of the blood-brain barrier: therapeutic avenues. *FASEB J.* 36:e22107. 10.1096/fj.202101297R 34939700

[B133] Sridharan A. Pehar M. Salamat M. S. Pugh T. D. Bendlin B. B. Willette A. A. (2013). Calorie restriction attenuates astrogliosis but not amyloid plaque load in aged rhesus macaques: a preliminary quantitative imaging study. *Brain Res.* 1508 1–8. 10.1016/j.brainres.2013.02.046 23473840 PMC3652278

[B134] Stansfeld S. Clark C. Bebbington P. E. King M. Jenkins R. Hinchliffe S. (2016). *Common Mental Disorders.* Leeds: NHS Digital.

[B135] Stubbs B. J. Koutnik A. P. Goldberg E. L. Upadhyay V. Turnbaugh P. J. Verdin E. (2020). Investigating ketone bodies as immunometabolic countermeasures against respiratory viral infections. *Med*. 1 43–65. 10.1016/j.medj.2020.06.008 32838361 PMC7362813

[B136] Sudo N. Chida Y. Aiba Y. Sonoda J. Oyama N. Yu X. N. (2004). Postnatal microbial colonization programs the hypothalamic–pituitary–adrenal system for stress response in mice. *J. Physiol.* 558 263–275. 10.1113/jphysiol.2004.063388 15133062 PMC1664925

[B137] Van der Auwera I. Wera S. Van Leuven F. Henderson S. T. (2005). A ketogenic diet reduces amyloid beta 40 and 42 in a mouse model of Alzheimer’s disease. *Nutr. Metab.* 2 1–8. 10.1186/1743-7075-2-28 16229744 PMC1282589

[B138] Veech R. L. Chance B. Kashiwaya Y. Lardy H. A. Cahill Jr, G. F (2001). Ketone bodies, potential therapeutic uses. *IUBMB Life* 51 241–247. 10.1080/152165401753311780 11569918

[B139] Wahl D. Coogan S. C. Solon-Biet S. M. de Cabo R. Haran J. B. Raubenheimer D. (2017). Cognitive and behavioral evaluation of nutritional interventions in rodent models of brain aging and dementia. *Clin. Interv. Aging* 12:1419. 10.2147/CIA.S145247 28932108 PMC5598548

[B140] Wang F. Cao Y. Ma L. Pei H. Rausch W. D. Li H. (2018). Dysfunction of cerebrovascular endothelial cells: prelude to vascular dementia. *Front. Aging Neurosci.* 10:376. 10.3389/fnagi.2018.00376 30505270 PMC6250852

[B141] Witte A. V. Fobker M. Gellner R. Knecht S. Flöel A. (2009). Caloric restriction improves memory in elderly humans. *Proc. Natl. Acad. Sci. U.S.A.* 106 1255–1260. 10.1073/pnas.0808587106 19171901 PMC2633586

[B142] Wu Y. J. Zhang L. M. Chai Y. M. Wang J. Yu L. F. Li W. H. (2016). Six-month efficacy of the Ketogenic diet is predicted after 3 months and is unrelated to clinical variables. *Epilepsy Behav.* 55 165–169. 10.1016/j.yebeh.2015.12.008 26785223

[B143] Yanai S. Okaichi Y. Okaichi H. (2004). Long-term dietary restriction causes negative effects on cognitive functions in rats. *Neurobiol. Aging* 25 325–332. 10.1016/S0197-4580(03)00115-5 15123338

[B144] Yao J. Irwin R. W. Zhao L. Nilsen J. Hamilton R. T. Brinton R. D. (2009). Mitochondrial bioenergetic deficit precedes Alzheimer’s pathology in female mouse model of Alzheimer’s disease. *Proc. Natl. Acad. Sci. U.S.A.* 106 14670–14675. 10.1073/pnas.0903563106 19667196 PMC2732886

[B145] Yin Z. Raj D. D. Schaafsma W. van der Heijden R. A. Kooistra S. M. Reijne A. C. (2018). Low-fat diet with caloric restriction reduces white matter microglia activation during aging. *Front. Mol. Neurosci.* 11:65. 10.3389/fnmol.2018.00065 29593493 PMC5857900

[B146] Yudkoff M. Daikhin Y. Nissim I. Lazarow A. Nissim I. (2001). Ketogenic diet, amino acid metabolism, and seizure control. *J. Neurosci. Res.* 66 931–940. 10.1002/jnr.10083 11746421

[B147] Zhang B. Wang H. E. Bai Y. M. Tsai S. J. Su T. P. Chen T. J. (2021). Inflammatory bowel disease is associated with higher dementia risk: a nationwide longitudinal study. *Gut* 70 85–91. 10.1136/gutjnl-2020-320789 32576641

[B148] Zheng P. Zeng B. Liu M. Chen J. Pan J. Han Y. (2019). The gut microbiome from patients with schizophrenia modulates the glutamate-glutamine-GABA cycle and schizophrenia-relevant behaviors in mice. *Science Adv.* 5:eaau8317. 10.1126/sciadv.aau8317 PMC6365110 30775438

[B149] Zwickey H. Horgan A. Hanes D. Schiffke H. Moore A. Wahbeh H. (2019). Effect of the anti-inflammatory diet in people with diabetes and pre-diabetes: a randomized controlled feeding study. *J. Restor. Med.* 8:e20190107. 31179163 10.14200/jrm.2019.0107 PMC6550471

